# Upregulation of XIAP promotes lung adenocarcinoma brain metastasis by modulating ceRNA network

**DOI:** 10.3389/fonc.2022.946253

**Published:** 2022-08-05

**Authors:** Yingjing Wang, Lu Shen, Geng Li, Jiayi Chen, Rong Ge

**Affiliations:** ^1^ Department of Diagnosis, Ningbo Diagnostic Pathology Center, Ningbo, China; ^2^ Hwa Mei Hospital, University of Chinese Academy of Sciences, Ningbo, China

**Keywords:** lung adenocarcinoma, brain metastasis, XIAP, has-miR-338-3P, immune infiltration

## Abstract

Dysregulation of XIAP has been shown to affect the progression of a variety of cancers, including lung adenocarcinoma (LUAD). However, the function and mechanisms of XIAP in lung adenocarcinoma with brain metastasis (LUAD-BM) remains poorly understood. In this study, we analyzed the differential mRNA of 58 lung adenocarcinomas samples and 28 lung adenocarcinomas with brain metastases in GEO database. 191 differentially expressed mRNAs were significantly associated with immune response, the proliferation of the immune cell, cell-cell adhesion. Subsequent analyzed by lasso and SVM found that XIAP was significantly elevated in LUAD-BM and significantly associated with LUAD grade and metastasis. Then we constructed a molecular regulatory network of ncRNA-miRNA-mRNA ceRNA by Cystoscope based on the correlation obtained from Starbase. It was found that SBF2-AS1 or RUNDC3A-AS1, has-miR-338-3p and XIAP may have a regulatory relationship. Furthermore, we also initially found that XIAP was closely correlation with T cells, B cells, Mast cells, macrophages, and dendritic cells. In conclusion, we found that XIAP was significantly higher expressed in LUAD-BM compared with LUAD without brain metastasis, suggesting that XIAP may play an important role in the future prediction and clinical treatment of LUAD-BM.

## Introduction

Globally, lung cancer is the malignant tumor with the highest incidence rate and poor clinical treatment effect. About 2.2 million new cases were reported worldwide in 2020, which accounts for about 11.4%, and the number of lung cancer-related deaths was 1.8 million, accounting for 18% (1). Approximately, 40-50% of lung cancer patients have brain metastases (BM), particularly with lung adenocarcinoma (LUAD) accounting for about 11% (2). However, the efficiency of drug transport through the blood-brain barrier is low, and an effective BM treatment remains a daunting challenge (3). Studies have shown that there may be additional potential carcinogenic changes in BM (4). Therefore, a better understanding of the mechanism of BM and the discovery of new genomic signatures will be a significant milestone in the treatment of BM.

The X-linked inhibitor of apoptosis protein (XIAP) belongs to the inhibitor of apoptosis proteins (IAP) family. Its inhibition of cell apoptosis is principally by interfering with the function of caspase-3/-7/-9, also, it participates in cell autophagy, necrosis, and the regulation of homeostasis (5). XIAP is highly expressed in 60 human tumor cell lines of the National Cancer Institute (6). Besides the research on the anti-apoptotic function of XIAP, some scholars have found that XIAP affects the invasion and lung metastasis of bladder cancer by regulating ERKS (7). Additionally, XIAP can attenuate RhoGDIα SUMOylation at lys-138 to regulate the invasion of colon cancer cells (8). However, the influence and molecular mechanism of lung cancer metastasis have not been explored.

The ceRNA hypothesis describes the competing activities of some RNAs at the common binding sites of targeted miRNAs, thereby achieving the function of regulating miRNAs. The ceRNA network links the functions of protein-coding mRNAs and thus the functions of non-coding RNAs, of which lncRNAs are crucial in the regulation of gene expression (9, 10). Studies have found that lncRNA CRNDE reduces XIAP protein levels by negatively regulating miR-186 (11). In the current study, we compared the difference in the expression of mRNA between lung cancer and lung cancer patients with brain metastases. Fortunately, the combined Lasso regression analysis and SVM regression algorithm revealed that XIAP was significantly high in patients with lung cancer brain metastases. Furthermore, small sample tests and ceRNA network predictions were performed to explore the clinical value of XIAP as a signature gene for brain metastases and to study the correlation with the infiltration of immune tumors.

## Materials and methods

### Eligible dataset filtering

Two mRNA expression profiles (GSE14108, GSE10072) were acquired from the GEO platform (http://www.ncbi.nlm.nih.gov/geo), a public repository containing a high-throughput genomics database (12). 58 LUAD samples and 28 lung adenocarcinomas with brain metastases (LUAD-BM) were used as target datasets. The external validation profiles were obtained from GSE126548 and it contains matched 3 LUAD tumors and 3 LUAD-BM samples. The datasets were standardized by fragment per kilobase million (FPKM).

### Identification of differentially expressed BM-related genes

The significant differential genes (DEGs) were identified using the “limma” package based on the R 4.1.2 environment (13). Log Fold Change (log_2_FC) >2 and the adjusted P <0.05 were considered to be statistically significant. Subsequently, BM-related differential genes were screened using LASSO and SVM-RFE regression analysis, respectively, and the Venn diagrams were drawn. Finally, the expression of BM-related differential genes was verified in the GSE126548 dataset to ensure the accuracy of screening.

### Functional enrichment analysis

Based on the DEGs screening, we used the “clusterProfiler” package to complete the Kyoto Encyclopedia of Genes and Genomes (KEGG) and the Gene Ontology (GO) enrichment analysis. The p-value and q-value less than 0.05 were considered to be significantly enriched. In addition, we used the STRING version11.5 (https://www.string-db.org/) to construct a PPI network diagram and performed the KEGG analysis (14).

### Prediction of the lncRNA-miRNA-mRNA ceRNA network

The mRNA and lncRNA data and related clinical information were obtained from UCSC Xena (https://xenabrowser.net) (15). The miRNA expression profiles were downloaded from TCGA (https://portal.gdc.cancer.gov/) and the differential miRNAs and lncRNAs were screened using the “limma” package. Subsequently, the upstream miRNAs and lncRNAs which interact with XIAP were obtained from the starbase database (https://starbase.sysu.edu.cn) (16). Starbase is an open-source platform for studying the miRNA-lncRNA, miRNA-mRNA, and lncRNA-RNA interactions from CLIP-seq, degradome-seq, and RNA-RNA interactome data. In order to improve the prediction accuracy, we included 7 datasets including PITA, RNA22, miRmap, miRanda, PicTar, TargetScan and Pan-cancer. The “program Number≥2” were regarded as being significantly significant predictions. Furthermore, the “limma” package was used to analyze the interactions among the lncRNA, miRNA, and mRNA. The Correlation coefficient≥2, and P-value<0.001 were considered to be significant. Cytoscape 3.7.1 was used for the visualization of the miRNA-mRNA and lncRNA-miRNA-mRNA network (17).

### The infiltration of the immune microenvironment

CIBERSORT is a widely used immune infiltration analysis tool and provide an estimation of the abundances of member cell types in a mixed cell population, using gene expression data (18). So we used CIBERRSORT to quantify the proportion of 22 immune cells in patients with LUAD or LUAD-BM (19). The correlations between immune cells were plotted using the “corrplot” package. The correlation between DEGs and the immune cells was then analyzed using “limma”, “reshape2”, “ggpubr”, and “ggExtra” packages.

### Statistical analysis

The Spearman and distance correlation analyses were performed to generate the correlation coefficients. The comparative analysis of the two groups was performed using the Wilcox test. All statistical analyses were performed in R 4.1.2, GraphPad Prism 8, and SPSS20. All statistical P values are two-sided and *a priori* P < 0.05 represents statistical significance.

## Result

### Analysis of the genetic differences between LUAD and LUAD-BM

**Figure 1:**
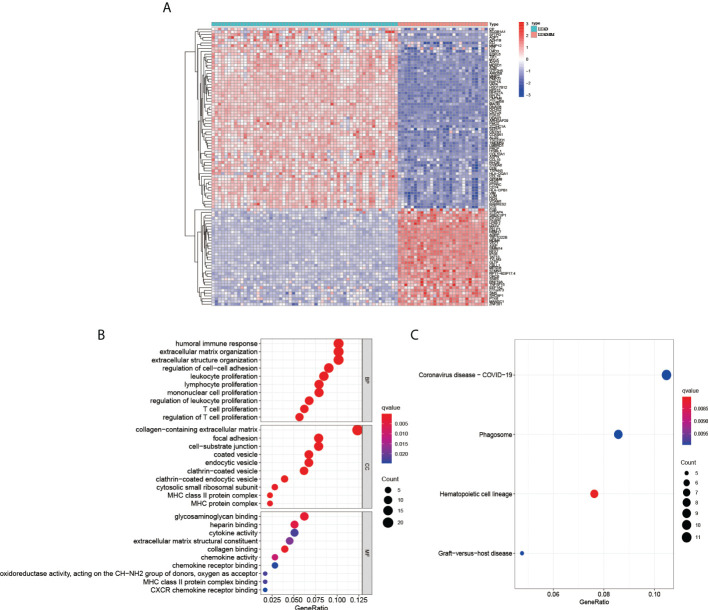
Analysis the differential genes between LUAD and LUAD-BM. **(A)** Heatmap of LUAD and LUAD-BM (|log2FC|≥2, p < 0.05). The GO biological function analysis **(B)** and KEGG pathway analysis **(C)** of DEGs.

To perform a preliminary exploration of the genetically significant differences between LUAD and LUAD-BM, we increased the screening criteria to |log2FC|≥2 to screen for differential genes. Then, we initially screened out 191 significant DEGs, including 35 up-regulated genes and 156 down-regulated genes (**Figure 1A**). The Go enrichment analysis showed that the DEGs were significantly associated with biological functions like immune response, the proliferation of the immune cell, cell-cell adhesion, etc. (**Figure 1B**). The KEGG pathway enrichment suggests that DEGs were correlated with Coronavirus disease-COVID-19, phagosome, hematopoietic cell lineage, and graft−versus−host disease (**Figure 1C**).

### Identified the most relevant genes for LUAD-BM

**Figure 2:**
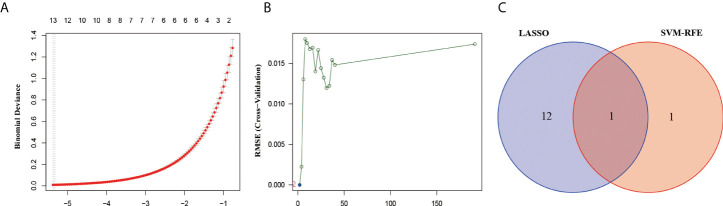
Screened the most relevant genes for LUAD-BM. **(A)** Ten-time cross-validation for DEGs in the LASSO. **(B)** The Root Mean Square Error (RSME) curve of DEGs in SVM-REF. **(C)** The intersection genes selection between LASSO and SVM-RFE algorithms.

To further explore the markers associated with BM, we first found 13 genes that were significantly associated with LUAD-BM using Lasso linear regression models, including DCHS2, AGRP, CFL1, TMC7, TBC1D22B, RPS27, PTPN9, RPS29, RPS15A, RBM17, XIAP, HERC1, and TXLNG (**Figure 2A**). Subsequently, the SVM-RFE algorithm identified two genes to be significantly associated with LUAD-BM, namely MDM4 and XIAP (**Figure 2B**). Interestingly, both methods detected XIAP as a signature gene (**Figure 2C**).

### External dataset validation

**Figure 3:**
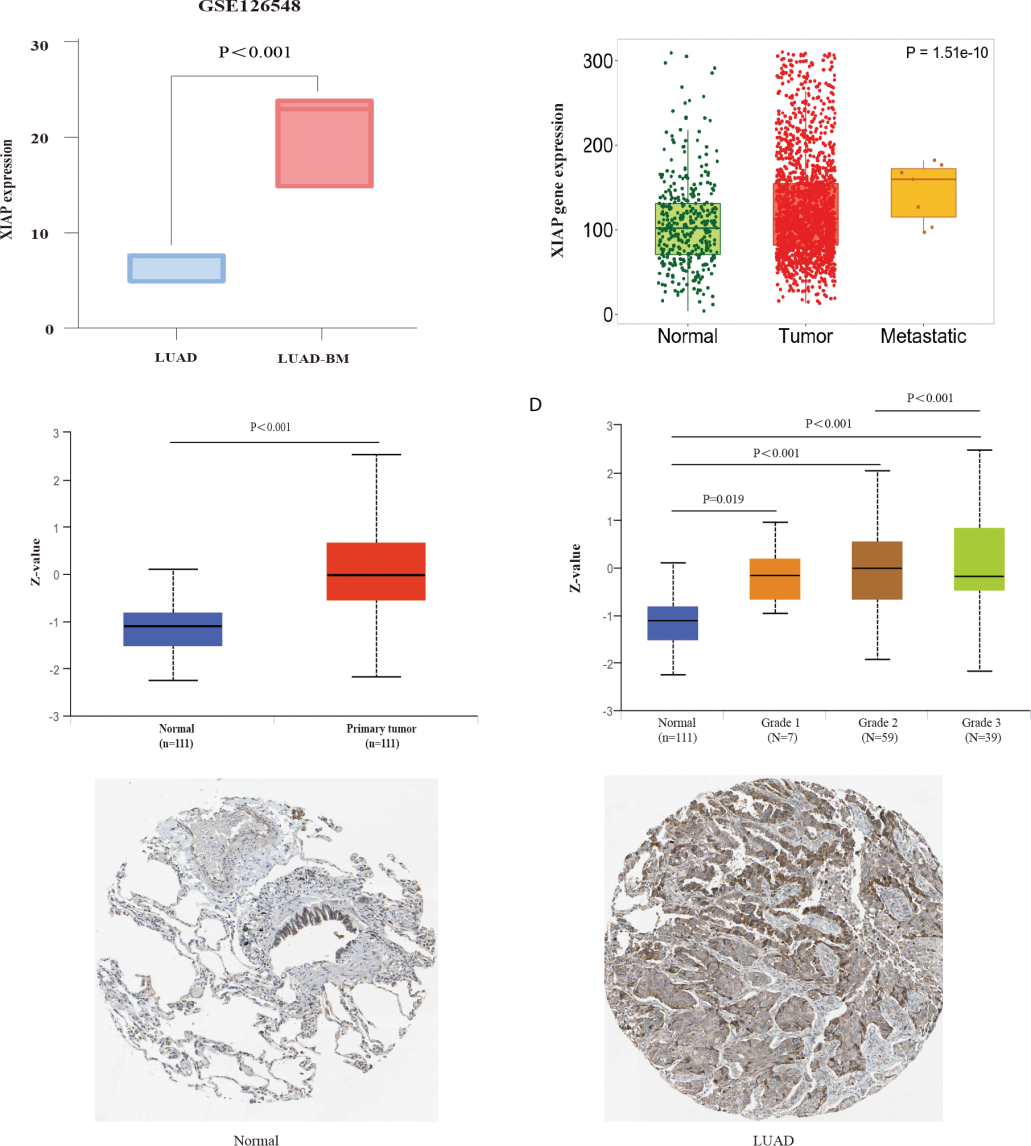
External data validation of XIAP expression in LUAD and LUAD-BM. **(A)** The expression of XIAP for LUAD and LUAD-BM in GSE126548. **(B)** The relationship of XIAP and LUAD metastasis in TNM plot. **(C, D)** upregulated expression of XIAP protein in LUAD tissue was associated with tumor grade. **(E)** The Immunohistochemistry showed that XIAP protein was increased in LUAD tumor than normal tissue in HPA database. P < 0.05 indicated significant difference.

Furthermore, we used the GSE126548 as the external validation data and found that the XIAP mRNA is also improved significantly in the LUAD-BM sample (**Figure 3A**), suggesting that XIAP can predict BM accurately. The high expression of XIAP mRNA in LUAD and metastasis was also found in the expression of differential gene analysis in the tumor, normal, and Metastatic Tissue (TNMplot, https://tnmplot.com) (**Figure 3B**).

Meanwhile, we also used the UALCAN database to explore the up-regulated XIAP protein in LUAD tissue and grade 3 patients (**Figures 3C, D**). Furthermore, the HPA database was also compared with normal lung tissue, and the XIAP was found to be more expressive in LUAD (**Figure 3E**).

### Function analysis of XIAP

**Figure 4:**
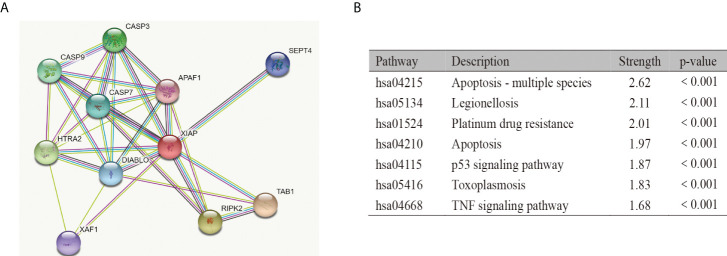
Function analysis of XIAP. The protein-protein interaction **(A)** and KEGG pathway analysis **(B)** of XIAP.

The STRING database was used to screen 10 genes that were highly related to XIAP (Score>0.9). The PPI network suggested that XIAP has a direct association with TAB1, RIPK2, HTRA2, CASP3, CASP9, CASP7, DIABLO, SEPT4, XAF,1, and APAF1 (**Figure 4A**). Furthermore, the enrichment of the KEGG signaling pathway showed that the expression of XIAP protein was correlated with Apoptosis - multiple species, Legionellosis, Platinum drug resistance, Apoptosis, p53 signaling pathway, Toxoplasmosis, and TNF signaling pathway (p-value<0.001 **Figure 4B**).

### Constructed and identification of the lncRNA-miRNA-mRNA axis

**Figure 5:**
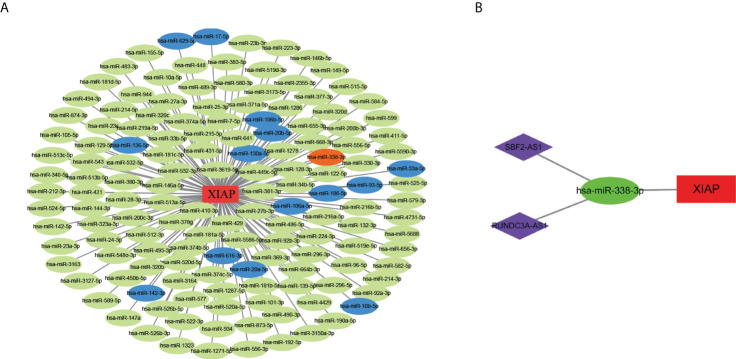
The construction of lncRNAs-miRNAs-XIAP network. **(A)** The miRNAs and XIAP network in LUAD. Green nodes indicated significant differential miRNAs associated with XIAP in TCGA database. Blue nodes suggested the miRNAs positively correlated with XIAP in Starbase (The Correlation coefficient ≥ 2, and P-value < 0.001), while orange node indicate negatively. **(B)** the ceRNA network of SBF2-AS1, RUNDC3A-AS1, hsa-miR-338-3p, and XIAP.

To construct the lncRNA-miRNA-mRNA network, we initially identified the significant differential lncRNAs and miRNAs by R software and produced the target relationship between lncRNAs and miRNAs. The result showed that 146 miRNAs were closely correlated with XIAP mRNA. Secondly, 15 target miRNAs of XIAP were acquired from the starbase database, in which hsa-miR-338-3p was negatively correlated with XIAP (**Figure 5A**). Subsequently, we downloaded the 132 lncRNAs associated with hsa-miR-338-3p and overlapped with the result obtained from R. Finally, we got the ceRNA network of SBF2-AS1, RUNDC3A-AS1, hsa-miR-338-3p, and XIAP was drawn with Cytoscape 3.7.1 (**Figure 5B**).

**Figure 6:**
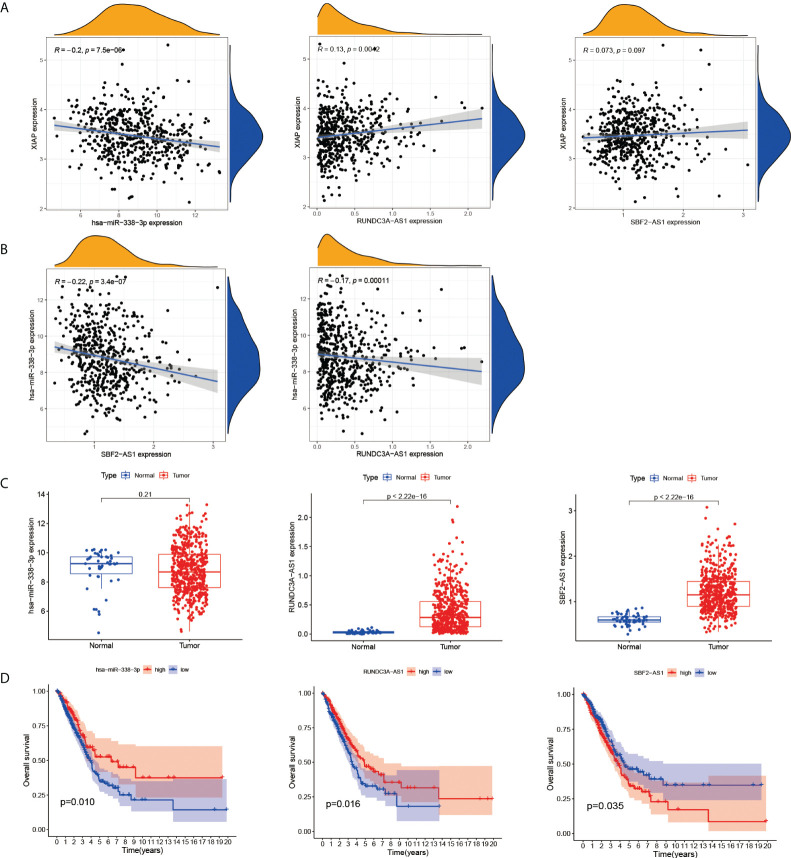
The correlation of XIAP, has-miR338-3p, SBF2-AS1 and RUNDC3A-AS1. **(A)** SBF2-AS1 and RUNDC3A-AS1 were positively associated with XIAP, and hsa-miR338-3p was negatively. **(B)** The relationship of SBF2-AS1or RUNDC3A-AS1 with hsa-miR338-3p. **(C)** The expression of has-miR338-3p, SBF2-AS1 and RUNDC3A-AS1 in LUAD and normal tissues. **(D)** The relationship of has-miR338-3p, SBF2-AS1 and RUNDC3A-AS1 and OS, P < 0.05 indicated significant difference.

The results showed that hsa-miR-338-3p expression was negatively correlated with XIAP, and RUNDC3A-AS1 and SBF2-AS1 were positively correlated with XIAP (**Figure 6A**). And SBF2-AS1 and RUNDC3A-AS1 expression decreased as has-miR-338-3p expression decreased (**Figure 6B**). Simultaneously, we linked the gene expression data with the clinical information, which demonstrated that RUNDC3A-AS1 and SBF2-AS1 have a highly significant expression in LUAD (**Figure 6C**) and can be a good biomarker for prognosis (**Figure 6D**). However, there was no significant difference in the hsa-miR-338-3p between the normal group and the LUAD.

### Comparison of the immune function

**Figure 7:**
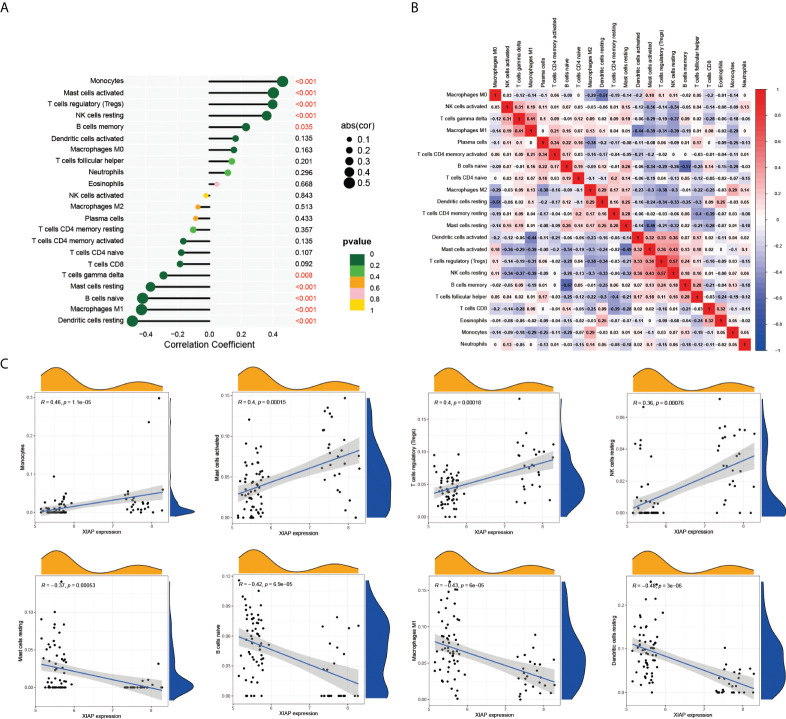
XIAP-related immune infiltration. **(A)** The relationship of 22 tumor infiltrating immune cells with LUAD-BM in CIBERSORT. **(B)** The proportions of different tumor infiltrating immune cell subpopulations were weakly to moderately correlated. **(C)** Monocytes, activated mast cells, T cell regulatory, and the resting NK cells were positively correlated with XIAP. In contrast, Mast cell resting, B cell naive, macrophages M1, and the resting dendritic cells were negatively related XIAP.

Previous GO analysis showed that LUAD-BM was closely associated with immune response. In this paper, we further explored the role of the immune microenvironment in LUAD-BM by CIBERSORT. The result demonstrated that monocytes, activated mast cells, T cell regulatory, and the resting NK cells were positively correlated with LUAD-BM and XIAP. Mast cell resting, B cell naive, macrophages M1, and the resting dendritic cells were negatively related to LUAD-BM and XIAP (**Figures 7A, C**). Additionally, the correlation heatmap revealed that the proportions of resting NK cells and T cell regulatory (Tregs) were moderately correlated (**Figure 7B**). These results suggested that XIAP may play an important role in T cells, B cells, Mast cells, macrophages, and the regulation of dendritic cells.

## Discussion

XIAP is an important member of the IAP family with three baculoviral IAP repeat (BIR) motifs, one ubiquitin-associated (UBA) domain accompanied by a ring finger domain (20). Previous studies on XIAP were mostly related to apoptosis, and a few research results have demonstrated the role of XIAP in cell metastasis. Metastasis often heralds a lethal stage of epithelial malignancy with few available treatment options (21), especially in LUAD. Here, we found that the up-regulated expression of XIAP was related to LUAD-BM. Meanwhile, the expression of XIAP protein had a significant association with LUAD grade and metastasis. In addition, this study revealed that different tumor-infiltrating immune cells were correlated with XIAP in LUAD-BM, and constructed the SBF2-AS1 or RUNDC3A-AS1-has-miR-338-3p-XIAP network. Thus, findings from our studies give evidence to infer that XIAP could serve as an important biomarker for LUAD-BM.

Over half of lung cancer patients developed brain metastases, and the incidence is increasing yearly, also, the median survival time is less than 6 months (22). To understand the mechanisms of LUAD-BM, we analyzed two mRNA profiles containing 58 lung adenocarcinoma samples and 28 lung adenocarcinomas with brain metastases. The result showed that 191 genes had more than a 2-fold difference. Moreover, the likely important signaling pathways in LUAD-BM include immune response, the proliferation of the immune cell, cell-cell adhesion, etc. This may be related to the expression of a variety of adhesion molecules involved in immune response and inflammation (23). Subsequently, we screened out highly expressed XIAP mRNA in LUAD-BM by lasso regression and SVM-RFE analysis. We also observed that the expression of XIAP mRNA and protein were up-regulated in LUAD than in normal tissues, and significantly associated with LUAD grade and metastasis. The research on XIAP as a metastasis-promoting factor has been deepening in recent years. Notable examples include: the improvement of cell adhesion by CAV1-mediated XIAP recruiting to the α-integrin complex (24). Another research demonstrated that XIAP, and surviving cooperate to regulate the invasion of tumor cells and metastasis (25). Hong Zhang et al. found that XIAP-shRNA significantly inhibited cell migration and invasion through a non-small cell lung cancer (NSCLC) xenograft model (26). Ceramide analog LCL85 targeting XIAP and CIAP1 overcomes apoptosis-induced resistance in metastatic colon and breast cancer, thereby inhibiting metastasis *in vivo* (27). Then, researchers confirmed that XIAP enhanced the nucleolin-mediated Rho-GDIβ mRNA stability to promote bladder cancer cell invasion of the lung (7). And curcumin inhibits the expression of XIAP and significantly reduces the incidence of breast cancer metastasis to the lung in a human breast cancer xenograft model (28). However, the role of XIAP in brain metastasis has not been established. Inhibition of XIAP or Survivin enhances postradiotherapy cell survival in lung cancer cells H460 compared to controls (29). XIAP-targeted shRNA and celecoxib synergistically reduce the growth of NSCLC (26). And XIAP-mediated protection of H460 lung cancer cells against cisplatin (30). Given the growing number of studies linking XIAP to cancer, focus has shifted to the development of anti-XIAP drugs. Thus, the discovery of increased XIAP in LUAD-BM and the evaluation of the mechanisms are of tremendous importance to LUAD metastasis and clinical treatment.

An increasing number of studies have shown that lncRNAs may regulate the expression of the targeted mRNA by competing for the shared miRNAs (31). For instance, miR-185 specifically binds to XIAP to reduce glioma stability, a process that can be inhibited by CRNDE (11). The 3’UTR of XIAP can also function as a ceRNA, and decreased miR-29a-5p adsorbed FSCN1 increased motility of breast cancer cells. In this study, we analyzed the ana LUAD expression profiles to screed the differential expression of lncRNAs and miRNAs. By integrating the interaction between them, we found that miR expression was negatively correlated with XIAP, and RUNDC3A-AS1 and SBF2-AS1 were positively correlated with XIAP. In addition, SBF2-AS1 and RUNDC3A-AS1 expression decreased as has-miR-338-3p expression decreased. These studies suggested that SBF2-AS1 or RUNDC3A-AS1 might regulate the expression of XIAP target *via* has-miR-338-3p. Generally, miRNA is known to negatively regulate the expression of genes at the mRNA (32). A large number of studies have found that has-miR-338-3p inhibits the proliferation and invasion of lung cancer cells by targeting SOX4, IRS2 and AKT (33–35). Thereby, we have reason to believe that has-miR-338-3p plays an important role in lung cancer invasion. The association of SBF2-AS1with has-miR-338-3p was reported, which is consistent with our predicted results. For example, SNF2-AS1 was showed to inhibit the proliferation and migration of clear cell renal cell carcinoma by inhibiting miR-338-3p targeted ETS1 (36). SBF2-AS1 can also affect the malignant phenotype of non-small cell lung cancer (NSCLC) by participating in miR-338-3p/ADAM17 axis (37). However, RUNDC3A-AS1 has not been extensively studied, and it was currently discovered that RUNDC3A-AS1 regulated the malignant progression of thyroid cancer and can promote lung metastasis by targeting miR-182-5p/ADAM9 (38, 39). New research has shown that miR-338-3p suppresses the metastasis of lung cancer by influencing the MAPK signaling pathway or targeting KIF2A (40, 41). In this study, we found that miR-338-3p was decreased with higher expression of XIAP, however, there was an increment in SBF2-AS1 and RUNDC3A-AS1. Thereby, SBF2-AS1 or RUNDC3CA-AS1 may become competitively bonded to miR-338-3p, which leads to the release of the inhibition of XIAP by miR-338-3p.

Another noteworthy finding in this research is the correlation between immune infiltration and LUAD-BM. The CIBERSORE analysis demonstrated that monocytes, activated mast cells, T cell regulatory, and NK cell resting were significantly upregulated in the LUAD-BM group, with an increase in the expression of XIAP. However, Mast cell resting, B cell naive, macrophages M1 and dendritic cells resting were downregulated. This correlation could indicate that XIAP plays an important role in naïve immune response, which is consistent with the findings of Anne C et al (42). However, further studies are still demanded to confirm our findings.

In summary, the up-regulated expression of XIAP was significantly associated with LUAD metastasis, especially in brain metastasis. This function may be closely related to the negative regulation of has-miR-338-3p by SBF2-AS1 or RUNDC3A-AS1. However, this study is limited by its dependence on bioinformatics analysis only, and needs further *in vivo* and *in vitro* studies to verify. In summary, XIAP could be considered a new candidate for therapeutic target in LUAD-BM.

## Data availability statement

Publicly available datasets were analyzed in this study. This data can be found here: GEO platform http://www.ncbi.nlm.nih.gov/geo;UCSC Xena (https://xenabrowser.net).

## Author contributions

Guarantor of integrity of the entire study: RG. Study concepts and design: RG and YW. Literature research and analysis: GL and JC. Data analysis and statistical analysis: YW and LS. Manuscript editing: YW. Final approval of manuscript: All authors. All authors contributed to the article and approved the submitted version.

## Funding

Medical science and technology program in Ningbo (2021Y28). The Project of Medical and Health Research Fund Project in Zhejiang Province (2022KY1185).

## Conflict of interest

The authors declare that the research was conducted in the absence of any commercial or financial relationships that could be construed as a potential conflict of interest.

## Publisher’s note

All claims expressed in this article are solely those of the authors and do not necessarily represent those of their affiliated organizations, or those of the publisher, the editors and the reviewers. Any product that may be evaluated in this article, or claim that may be made by its manufacturer, is not guaranteed or endorsed by the publisher.
